# Biological Responses to the Transitional Area of Dental Implants: Material- and Structure-Dependent Responses of Peri-Implant Tissue to Abutments

**DOI:** 10.3390/ma13010072

**Published:** 2019-12-22

**Authors:** Jung-Ju Kim, Jae-Hyun Lee, Jeong Chan Kim, Jun-Beom Lee, In-Sung Luke Yeo

**Affiliations:** 1 Department of Periodontology, Seoul National University School of Dentistry, Seoul 03080, Korea; freetist@gmail.com (J.-J.K.); perio_kjc@naver.com (J.C.K.); dent.jblee@gmail.com (J.-B.L.); 2 Department of Prosthodontics, One-Stop Specialty Center, Seoul National University Dental Hospital, Seoul 03080, Korea; jhlee.snudh@gmail.com; 3 Department of Prosthodontics, School of Dentistry and Dental Research Institute, Seoul National University, Seoul 03080, Korea

**Keywords:** abutment, dental implant, implant connection, marginal bone, peri-implantitis

## Abstract

The stability of peri-implant tissue is essential for the long-term success of dental implants. Although various types of implant connections are used, little is known about the effects of the physical mechanisms of dental implants on the stability of peri-implant tissue. This review summarizes the relevant literature to establish guidelines regarding the effects of connection type between abutments and implants in soft and hard tissues. Soft tissue seals can affect soft tissue around implants. In external connections, micromobility between the abutment and the hex component of the implant, resulting from machining tolerance, can destroy the soft tissue seal, potentially leading to microbial invasion. Internal friction connection implants induce strain on the surrounding bone via implant wall expansion that translates into masticatory force. This strain is advantageous because it increases the amount and quality of peri-implant bone. The comparison of internal and external connections, the two most commonly used connection types, reveals that internal friction has a positive influence on both soft and hard tissues.

## 1. Introduction

A dental implant is an artificial organ that replaces a missing natural tooth. Implants should be able to function properly in the human body [1]. Long-term stability should be predictable and in line with current trends toward increased life expectancy [2,3,4,5,6]. For dental implants to function properly for a long time in the oral cavity, they should experience neither mechanical nor biological complications, including those related to soft and hard tissues surrounding the implant [7,8,9,10,11].

The stability of the implant–abutment connection is an important factor affecting the long-term success of dental implants in clinical practice [12,13]. To prevent complications resulting from unstable implant–abutment connections, implant–abutment biomechanics are investigated theoretically and experimentally. In this review, internal friction connection and external hex-type connection implant–abutment joints are explored. Dental implants belonging to these two systems are currently the most widely used in clinical practice [13,14,15]. The nature of these connections is summarized, and their effects on tissues surrounding implants are analyzed for both soft and hard tissues.

## 2. Typical Dental Implant Connection Types

### 2.1. External Hex Connection: Butt-Jointed Interface

In external hex-type connections, the abutment is connected to the implant by an abutment screw. This connection is also called a butt joint interface because the flat surfaces on the top of the implant and the bottom of the abutment are in direct contact with each other. This external connection is stabilized entirely by fastening of the abutment screw. When torque is applied to the abutment screw connecting the implant and the abutment, the abutment screw is elongated, generating a preload. Screw dynamics play an important role in this interaction because the mechanism linking this connection is fundamentally dependent on the preload of the screw [16]. The preload acts as a clamping force on the implant–abutment complex, and provides stability to the connection [17].

The Brånemark implant (NobelBiocare, Zurich, Switzerland), the first commercially available screw-shaped implant, is a representative external connection-type implant. When masticatory force is applied to this type of implant, the vertical component of the masticatory force is supported by the top platform of the implant. The lateral component of the masticatory force is placed on the hex structure of the top of the implant and the abutment screw, and the rotational component of this force is resisted by the hex structure.

Previous biomechanical analyses indicate that the manufacturer’s recommended torque of 30–35 Ncm is not sufficient to prevent screw loosening [17,18]. Repeated tightening of the abutment screw is considered essential because of the preload loss in screw mechanics, which is the cause of screw loosening [17]. It is also helpful to use other methods in addition to screw tightening to maintain the stability of the implant–abutment connection. One option that makes this possible is the use of the frictional force generated between the interfaces.

### 2.2. Internal Friction Connection: Frictional Interface

In internal friction connections, stability is maintained by close contact between the inner surface of the implant and the outer surface of the abutment, in addition to the preload applied to the abutment screw. The tight contact between the abutment and implant creates frictional force, and this plays a major role in supporting the stability of the connection. Therefore, this type of connection is also called a friction-screw-retained connection.

**Figure 1:**
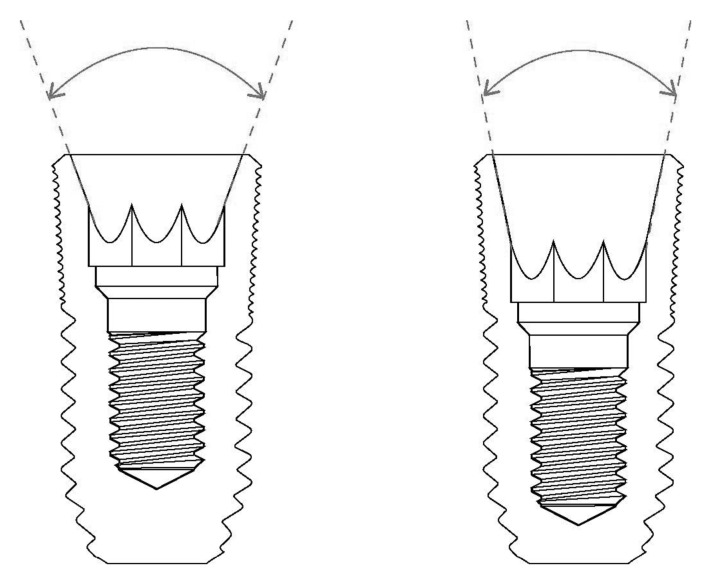
The degree of conical connection. A wider tapering angle (left) results in a more unstable connection for abutment mobility in internal connection-type implants.

The degree of tapering at the interface between the implant and the abutment is an important factor to consider when determining the abutment mobility of the internal conical connection. A wider tapering angle will result in a more unstable connection in this type of internal connection, although the possibility of implant fracture decreases [16,19]. In addition, the role of frictional forces in maintaining the stability of the connection is decreased, while the burden on the abutment screws is increased (Figure 1).

A systematic review reported that internal friction connection provides less microleakage at the implant–abutment interface than external hex connection type in both static and dynamic loading tests [20]. The presence of gap could lead to accumulation of bacteria and the phenomenon might affect the success rate of implants.

When an abutment is connected to an internal conical connection-type implant, the abutment is first brought into contact with the upper part of the implant inner wall [19,21]. As the implant at the top is slightly flared, it comes into greater contact with the abutment as the implant is combined. The abutment screw becomes slightly loosened when the abutment is completely combined with the implant by opening the implant wall. Because of the preload loss from such an abutment sinking and settling effect, repeated screw tightening is essential [18,22].

## 3. Soft Tissue Responses to Different Implant System Materials and Structures

### 3.1. The Soft Tissue Seal Theory

Humans are exposed to a variety of external environments involving external forces, ultraviolet rays, and microorganisms. Human skin is the first line of defense that protects the human body against these external stresses. When human skin is pierced, the resulting hole must be closed by the immune system and healing mechanisms.

Teeth are one of the few organs in the human body that are located across the skin. The root of the tooth is surrounded by alveolar bone, while the part that penetrates through the soft tissue is in contact with epithelial tissue and/or the connective tissue of the mucosa [23]. Holes in the mucosa created by teeth are sealed by a special structure composed of epithelium and connective tissue [23]. An internal basal lamina and the hemi-desmosomes of epithelial tissue are attached to teeth, and a combination of dento-gingival fiber and cementum links teeth to the surrounding connective tissue. The holes connecting the inside and outside of the human body that contain teeth are secured by soft tissue attachments [23].

**Figure 2:**
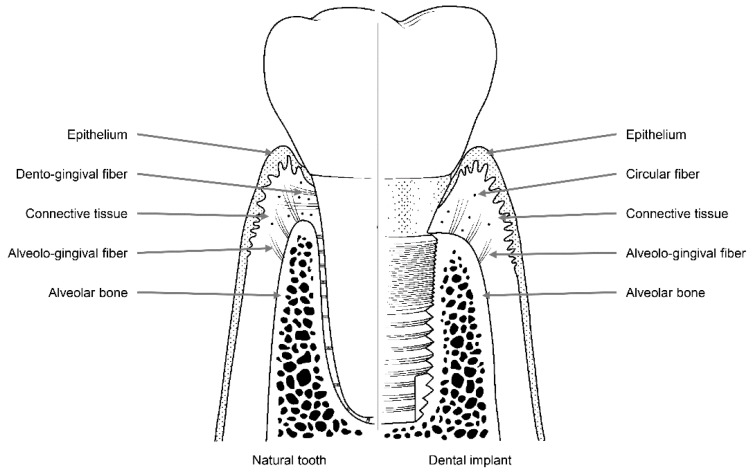
Soft and hard tissues with collagen fibers surrounding a natural tooth (**left**) and a dental implant (**right**).

Like natural teeth, dental implants are an artificial organ located in a hole on the surface of the body, and the hole is sealed via a mechanism similar to the one that seals the holes around natural teeth using soft tissues. However, these sealing mechanisms are not identical to each other. Dental implants are in contact with the alveolar bone or soft tissue (epithelial and connective tissue) of the transition area (Figure 2) [24]. Fibers in the connective tissue attached to the abutment mainly run parallel to the surface and are circular in shape, whereas dento-gingival fibers, such as Sharpey’s fibers, are attached vertically to the cementum in natural teeth (Figure 1) [24].

A scanning electron microscopy study showed that there are two distinct layers in the area within 200 μm of where the implant abutment comes into contact with the connective tissue. The inner 40 μm of this layer contains multiple fibroblasts that are attached directly to the surface of the abutment, while the outer 160 μm of this layer contains numerous collagen fibers [25]. Hence, the attachment of connective tissue to the abutment is maintained by fibroblasts attached to the titanium surface of the implant, and the elasticity of circular collagen fibers. For this reason, compared with the attachments of natural teeth, which involve direct attachment to connective tissue, attachments formed by connective tissue around an implant abutment are weaker.

Epithelial tissue is attached to the implant abutment via an internal basal lamina and hemi-desmosomes in a manner similar to the attachment of natural teeth [26,27,28,29]. However, epithelium attachment includes the internal basal lamina and hemi-desmosomes formed only in the lower part of the peri-implant epithelium around the implant abutment, whereas in natural teeth, these attachments are widely distributed throughout the junctional epithelium–tooth interface [30,31,32]. Thus, epithelial adhesions that form around the implant abutment are more vulnerable than those that form around natural teeth owing to their limited area of distribution.

In summary, both epithelial and connective tissues are more weakly attached to implant abutments than to natural teeth. Hence, holes on the surface of the human body that contain implants are more vulnerable to containment failure. This mechanism of blockade by soft tissue is called a ‘soft tissue seal’ [30,33].

### 3.2. Attachment of Soft Tissue

The stability and immobility of soft tissue attachments in contact with the implant abutment are important factors that affect the long-term prognosis of the implant [30,33]. If the soft tissues around the implant are deformed owing to the movement of the lip, cheek, tongue, or jaw, the weak soft tissue seal surrounding the implant may be destroyed. This allows microbes to penetrate through the damaged mucosal seal, increasing the likelihood of disease around the implant [30,33]. Therefore, a stable soft tissue seal is essential to prevent microbial invasion and peri-implant disease [33,34,35].

**Figure 3:**
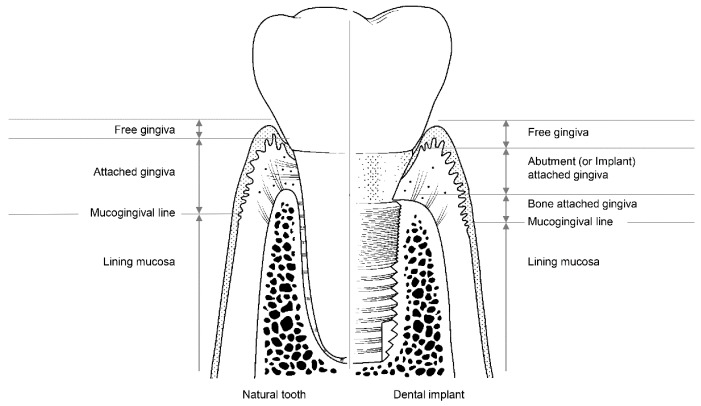
The structure of soft tissue around a natural tooth (**left**) and a dental implant (**right**).

The soft tissues around natural teeth are separated into the lining mucosa and masticatory mucosa, of which the masticatory mucosa is composed of the free and attached gingiva (Figure 3). The attached gingiva is in permanent and intimate contact with the surface of the enamel, the cementum, and the alveolar bone, thereby immobilizing the soft tissue [23]. It is, therefore, possible to retain the firmness and health of the mucosal seal by preventing it from detaching.

Among the soft tissues surrounding an implant, the attached gingiva differs from those surrounding natural teeth [24]. The gingiva attached to the peri-implant can be divided into two parts: bone-attached and abutment (or implant)-attached gingiva (Figure 3). The bone-attached gingiva is identical to the corresponding tissue in natural gums, and rigidly immobilizes the soft tissue. However, as mentioned above, the abutment- (or implant)-attached gingiva more weakly adheres than the attachment gingiva that surrounds natural teeth [24]. Owing to the weak structure of the abutment- (or implant)-attached gingiva, the width of the bone-attached gingiva around the implant should be wide enough to prevent mucosal mobility, and thereby prevent the occurrence of peri-implant disease due to the collapse of the mucosal seal [24,33].

### 3.3. Disruption of the Soft Tissue Seal

The soft tissue seal is mainly destroyed via two mechanisms; instability of the peri-implant mucosa or the implant-abutment assembly. If there is no bone-attached gingiva around the implant, the soft tissue can become mobile, and the mucosal seal will inevitably rupture. When the mucosal seal is destroyed, bacteria can penetrate the internal environment through the transmucosal rupture site, potentially leading to peri-implant disease [36]. Therefore, because of the weakness of the soft tissue attachment, it is important to ensure there is sufficient bone-attached gingiva around the implant. Thus, a plan for implant surgery should be established to maintain the bone-attached gingiva following implant placement.

Instability in the connection between the implant and the abutment can also lead to the disruption of the soft tissue seal, which may present as mobility of the abutment or the implant. Mobility of the implant occurs only when osseointegration fails, but mobility of the abutment can occur as a result of various causes, including fractures of the abutment or implant, even following successful osseointegration. The most common cause of abutment mobility is the loosening of the abutment screw that connects it to the implant. Elongation and loosening of the screw caused by the lateral force of the masticatory load are more frequently observed in external connection-type implants than in internal connection-type implants [15].

**Figure 4:**
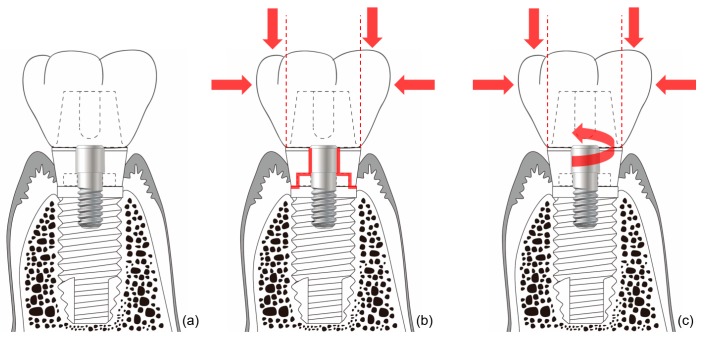
Schematic illustration of the ‘soft tissue seal’ theory. Micromobility of the abutment in external connection-type implants disrupts the surrounding soft tissue seal, which plays an important role in preventing external irritants from penetrating into the body. (**a**) External connection-type implant. (**b**) Parts experiencing the greatest stress (red lines) from eccentric forces (red arrows). (**c**) Abutment screw-loosening (red rotated arrow) by eccentric forces (red arrows).

In external-type implants, there is slight machining tolerance around the hex component, resulting in micromobility in the abutment. This external hex connection is, therefore, a mobile structure. Thus, most occlusal forces are concentrated on the abutment screw. This micromobility is likely to cause disruption of the soft tissue seal, bacterial infiltration, and peri-implant disease [37] (Figure 4). Implant systems with an external hex connection were originally developed for the mandibular restoration of a completely edentulous patient wearing a maxillary complete denture. The occlusal force in such patients is weak, and this mobile connection is able to bear this weak masticatory force with few screw loosening events or breakdown of the soft tissue seal. However, such unstable implant–abutment connections can provoke severe peri-implant problems for a partially edentulous patient with antagonistic natural teeth and a strong occlusal force. To reduce the micromobility of the abutment, the external hex must be lengthened, and the machining tolerance between the male and female components must be minimized. Furthermore, it may be possible to fabricate an abutment screw using a stronger material, or by widening the platform size of the top of the implant to minimize the force concentrated on the abutment screw. In addition, when the abutment is fastened to the implant, applying higher torque may also be employed to increase the stability of the connection. However, none of these methods can completely eliminate the micromobility of the abutment in external connection-type implants.

In internal connection-type implants, the abutment–implant connection is firm owing to a process similar to cold-welding. Unlike external connections, in which the occlusal force is concentrated mainly on the abutment screw, the occlusal force is transmitted to the implant inner wall through the abutment–implant connection in internal connection-type implants. Therefore, less screw loosening occurs in this type of implant. In addition, the abutment–implant contact area is wider internal connection-type implants, and this prevents stress from concentrating at specific sites such as the abutment screw, and contributes to the stability of the soft tissue seal.

### 3.4. Submerged and Nonsubmerged Implants

Depending on whether the top of the implant is at the alveolar bone or gingival level, implants can be divided into submerged-type and nonsubmerged-type. For submerged implants, the soft tissue seal is formed at the abutment area, rather than at the neck part for the nonsubmerged type. Histologically, there are no significant differences in the degree and pattern of soft tissue attachment to implants between submerged and nonsubmerged types [38]. When nonsubmerged implants are placed at the proper vertical position of the alveolar bone, the mobility of the abutment does not interfere with the soft tissue seal, or cause marginal bone resorption, because the interface between the abutment and the implant is positioned outside the soft tissue [34,35].

When this design was first developed, microgap bacteria residing between the abutment and implant were considered to be the cause of marginal bone resorption [39,40,41]. Therefore, a design was developed to export the position of this microgap outside the body [42]. However, such a design limits the customization of the patient’s emergence profile, resulting in difficulties in terms of long-term clinical results for aesthetically acceptable implant prostheses.

To maintain an adequate soft tissue seal, there must be a proper foundation of underlying bone. However, nonsubmerged-type implants can be susceptible to marginal bone resorption due to the concentration of stress at the top of the implant [43].

### 3.5. Materials for Abutment

Achieving soft tissue seals may also depend on the type of material used for the abutment. Abrahamsson et al. (1998) investigated the stability of soft tissue seals attached via gold alloy, dental porcelain, titanium, and aluminum oxide. In this study, marginal bone resorption occurred when seals were attached to surfaces composed of gold alloy and dental porcelain, because no soft tissue seal formed. However, when titanium and aluminum oxide were used, a soft tissue seal was achieved, and marginal bone was not absorbed [44]. Welander et al. (2008) also reported that epithelial adhesions receded around abutments attached via gold alloy [45]. Therefore, when using a UCLA abutment, the transmucosal area of the abutment is made of gold or dental porcelain, and a soft tissue seal may not be properly achieved, resulting in marginal bone resorption.

More recently, titanium and zirconia have been used as materials for abutments. One advantage of zirconia is that it is more aesthetic than titanium. While several studies show that there are no significant differences in the soft tissue seal between these two materials, zirconia has a lower fracture strength than titanium, and is thus more likely to be associated with mechanical complications [45,46,47]. In addition, adhesion of bacteria to titanium occurs less readily than to zirconia [48].

Recent advances in digital dentistry have made it possible to fabricate and restore implant-supported restorations in just a single visit. To this end, lithium disilicate and polymer-infiltrated ceramic network (PICN), which are easy to process on the day, are used as abutment material. These materials are used as implant abutments after cementing to the prefabricated titanium base with adhesive resin cement. Although little research has been conducted on the soft tissue seal of these new materials, Smallidge et al. have reported favorable epithelial cell growth on the PICN surface when PICN has a relatively low surface roughness (Ra < 0.254 µm) [49]. Another recent study reported that the surface roughness of lithium disilicate was smoother than that of PICN after the same polishing process with 6 μm diamond slurry, and the surface roughness of both of materials was unaffected by the ultrasonic scaling procedure [50]. Mehl et al. found that the adhesive resin joint connecting the titanium base and the abutment materials had neither influence on soft tissue anatomy nor on bone loss in the animal study [51]. In zirconia abutments, however, junctional epithelium was significantly shorter than titanium one-piece abutments [51]. Ongun et al. measured the mechanical properties of these hybrid-type abutments including titanium bases, showing that PICN had lower fracture resistance and weaker adhesion to resin cements compared with lithium disilicate ceramic [52]. Such mechanical failure of PICN hybrid-type abutments could also cause the soft tissue seal to break.

### 3.6. Detachment of Abutments

When the abutment becomes disconnected from a submerged-type implant, the soft tissue seal is broken, and microorganisms in the oral cavity can then penetrate into the tissues surrounding the implant [53]. This may result in the loss of marginal bone. Abrahamsson et al. reported that the absorption of marginal bone is doubled when the abutment is detached five times [54]. For these reasons, some clinicians have proposed the ‘one abutment-one time (OAOT)’ concept to prevent marginal bone loss [55,56]. Clinicians should be aware that during a prosthetic restoration procedure, or during the postrestoration maintenance period, it may be advantageous to minimize the process of removing the abutment.

A systematic review suggested that the OAOT protocol might preserve peri-implant bone loss and soft tissue changes for two reasons [57]. First of all, the micro-gap between abutment and implant could cause to bacterial leakage and micromotion, and this might lead to the inflammation of peri-implant soft tissue and bone resorption. The OAOT protocol could provide less micro-gap because healing or temporary abutments were installed by less preloading force (<10 Ncm) than final abutments (about 30 Ncm) [57]. Second, the OAOT protocol could reduce the disruption of the soft tissue seal around the implant abutment complex to avoid repeated the dis/reconnection of abutments [57].

### 3.7. Surface Modification of Abutments

Various technologies have been explored for improving the soft tissue seal at the transmucosal part of the abutment, including surface treatments, such as coating, machining, blasting, plasma spraying, etching, and laser processing [58].

Yang et al. reported that, when ultraviolet light is applied to surfaces, gingival fibroblasts proliferate more readily on surfaces made of zirconia [59]. Several studies showed that, when an abutment is laser-treated, the connective tissue is directly attached to the surface of the abutment by perpendicular fibers [60,61,62]. In addition, as the surface roughness of the transmucosal part of the abutment increases, the soft tissue seal improves. This may be because a rougher surface has a larger surface area to which the soft tissue can attach [63]. A previous study also suggested that hydrothermal treatment on titanium alloy could change minimally surface topography to enhance hydrophilicity [64]. Subsequently, the treatment contributed to the integration of epithelial cells to the surface and might facilitate the healthy epithelial tissue sealing around the transmucosal part of the implant [64].

The surface of the abutment can be modified to change the surface roughness or the free energy. While this may enhance the soft tissue seal, it can also increase plaque accumulation and the tendency for bacterial colonization to occur [65,66]. Thus, surface treatments applied to abutments should be carefully selected.

## 4. Hard Tissue Responses to Implant System Materials and Structures

### 4.1. The Bone Stimulation Theory

The soft and hard tissues surrounding the implant play complementary roles. Alveolar bone is a hard tissue that withstands the masticatory force applied to the implant, and serves to transmit it to the jawbone. The soft tissue, gingiva, and mucosa protect the alveolar bone from external irritants such as bacteria. The condition of the soft tissue is maintained by the underlying alveolar bone, and the alveolar bone is protected by the overlying soft tissue.

In general, after the restoration of an implant, between 1 and 1.5 mm of the marginal bone around the implant is absorbed during the first year owing to the application of occlusal force, and 0.2 mm is absorbed per year thereafter [67]. On the basis of the results of studies showing that marginal bone is steadily absorbed every year, it was thought that long-length implants should be beneficial for long-term predictability [67]. This was observed in long-term studies of the Brånemark system using an external connection-type implant, which is limited and inapplicable to some internal friction connections [14,68,69]. By contrast, several studies on a certain implant connection system reported that peri-implant marginal bone is increased by occlusal loading [68,69]. Because the use of long implants is likely to cause damage to important anatomical structures such as nerves, it may be more advantageous to insert short implants if warranted for long-term prognosis without marginal bone resorption.

In natural dentition, when periodontal disease occurs or a natural tooth is lost, the alveolar bone of the corresponding region is resorbed. However, when the natural tooth and the periodontal tissue are healthy, the alveolar bone is well-maintained. Except for a small amount of loss due to mechanical degeneration or aging, the alveolar bone should be well-maintained throughout life, as periodontal ligaments have been shown to transfer adequate stimulation to alveolar bone. Otherwise, disuse atrophy of the bone can occur. From this point of view, if the peri-implant bone is appropriately stimulated by the implant, the peri-implant marginal bone will not be resorbed, and the quantity and quality of the bone are likely to be preserved.

The Astra implant system (Dentsply Sirona, Charlotte, NC, United States) involves an internal friction connection type, and was the first connection system to embody this concept. The implant–abutment connection of this system improves the amount and quality of peri-implant bone by properly transmitting the masticatory force to the surrounding alveolar bone. This hypothesis was reported by Frost [70] and can be applied to alveolar bone as well as to other bones in the human body [70]. Hence, under conditions involving the application of appropriate strain to human bone, osteoblasts are activated, which increases the amount and quality of the bone.

**Figure 5:**
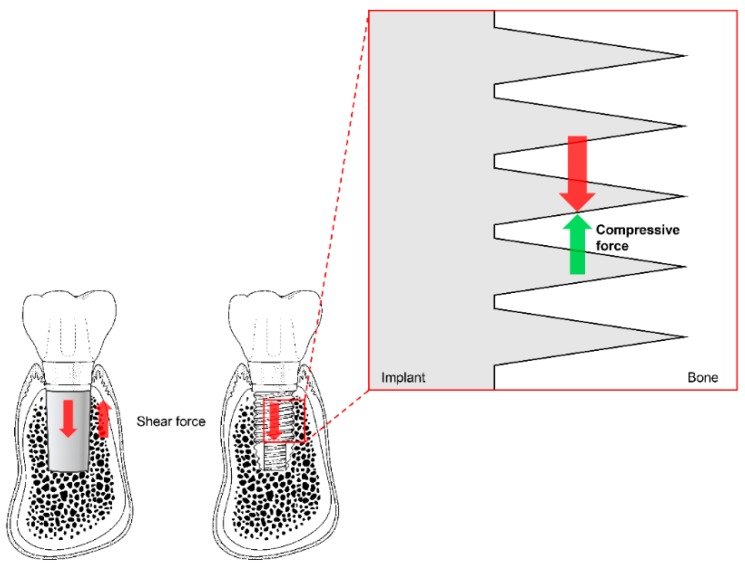
The thread function of non-threaded (**left**) and threaded (**right**) dental implants. Shear force (red arrows) is transformed into compressive force (green arrow) by the threads. Note the reduction of shear force due to the partial switch to compressive force in the magnified diagram (vertically and horizontally; not marked).

Before existing implant systems were widely used in clinical practice, various types and shapes of implants were developed, and most disappeared without displaying any ability to withstand mechanical or biological complications [71,72]. The Brånemark system was the first to achieve successful long-term predictable prognosis, and the consequential popularization of implants. Unlike previous plain-shaped systems, this system can deliver occlusal force to the alveolar bone because it has a thread on the implant surface [71,72]. The thread of the implant transforms the shear stress generated at the interface between the implant and the bone into compressive stress so that the appropriate stimulus can be transmitted to the bone (Figure 5). This stimulation is one of the factors that allows the bone to remain stable over the long term.

A disadvantage of these external connection-type implants is that the soft tissue seal formed around the abutment is destroyed by micromobility at the connection. The Brånemark implant represents the beginning of modern implants, and was the first system to perform well in clinical practice. However, marginal bone resorption was found to be caused by the destruction of the soft-tissue seal. To prevent such marginal bone loss, an Astra internal connection implant system was developed. This internal friction system maintains bone by applying appropriate stimulation to the bone around the implant without loss of the soft tissue seal around the implant.

### 4.2. Mechanism of Bone Stimulation

**Figure 6:**
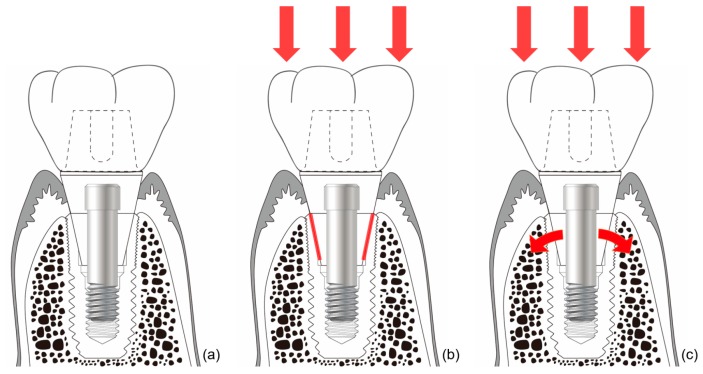
Schematic drawing of bone stimulation. When occlusal force is applied to an internal connection-type implant, the implant around the connection expands and stimulates the surrounding bone to induce bone proliferation. (**a**) Internal friction connection-type implant. (**b**) The occlusal force is transmitted to the implant through the conical connection parts (red lines). (**c**) The coronal expansion of the implant (curved red arrows) becomes the source of the strain that stimulates the alveolar bone.

The connection of the Astra implant system has an internal conical shape with a slope of 11 degrees. The occlusal force applied to the abutment is transmitted to the implant through this conical connection. The masticatory force delivered to the implant thus becomes the source of the stain to stimulate the alveolar bone [73]. Thus, when the abutment receives occlusal force and sinks downward, the conical opening of the implant is opened wider, and the bone around the implant is consequentially stimulated (Figure 6). This stimulation activates osteoblasts in the alveolar bone, thereby increasing the amount and quality of alveolar bone. This increase in alveolar bone can lead to a positive change in the results of clinical procedures. This reduces the need for invasive bone grafting or the placement of excessively long implants during implant surgery. This represents a positive result for both the patient and the surgeon.

### 4.3. Prevention of Hard Tissue Loss

The marginal bone loss that occurs in the mobile connection of external connection-type implants does not lead directly to the failure of the implant. However, it can cause considerable complications in the tissue surrounding the implant. In general, these external connection implants are known to lose marginal bone up to the second or third thread level of the implant [74]. This bone loss can alter the properties of the overlying soft tissue, resulting in a reduction in the attached gingiva. This weakens the soft tissue seal and increases the likelihood of bacterial invasion and peri-implantitis. A detailed periodical examination of the condition of tissues around the implant is thus essential for long-term success when external connection-type implants are used.

The issue of whether gingiva must be present for the health of natural teeth has been investigated. Clinically, even if the gingiva cannot completely wrap the tooth, it should be able to prevent the movement of the surrounding soft tissue from being transmitted to the free gingiva [75]. In other words, cheek, lip, and mandibular movements should not be transferred to the marginal gingiva. To achieve this, a certain amount of attached gingiva must be present to prevent the penetration of bacteria into the periodontal tissue [76].

This principle of natural dentition should also be borne in mind when considering implant restorations [77]. An appropriate amount of attached gingiva should be present, rather than free gingiva alone, to prevent microbial invasion into tissue surrounding the implant. According to the systematic review in 2018, if there is the insufficient attached gingiva around implant, the apically positioned flap with autogenous graft is suggested to increase the width of the gingiva [78]. However, it is not enough simply to increase the width of attached gingiva, because this surgical procedure is unable to alter the level of mucogingival junction [79]. Additional vestibuloplasty is, therefore, recommended to create “new” mucogingival junction by preventing the movement of the lips and cheeks from affecting the free gingiva.

In addition, abutment and restoration contour have been recently reported to have effects on marginal bone loss. An emergence angle of more than 30 degrees and a convex emergence profile may increase the risk of peri-implantitis [80]. The association between the restoration contour and the soft tissue seal remains uncertain. However, in order to prevent the bone loss, it is important to maintain the favorable soft tissue environment by adjusting the emergence angle, the emergence profile, the position of a restoration margin, and the removal of excess cement [80,81,82].

## 5. Summary

Dental implants should be capable of performing dental functions for a prolonged period of time. For this to be possible, the health of the surrounding soft and hard tissues is essential. Alveolar bone must be able to withstand masticatory force, and the overlying soft tissue should be able to protect the alveolar bone from external irritants.

The soft tissue seal is weaker around implants than around natural teeth. Thus, to avoid damage, the peri-implant soft tissue seal should not be mobile. Free and abutment-attached gingiva cannot prevent the destruction of the soft tissue seal that results from the movements of the lips and cheeks. Additionally, it is impossible to prevent all micromobility of the abutment in external connection-type implants. Hence, it is necessary to secure sufficient bone-attached gingiva, or use an implant system with extensive and deep connections, such as internal connection-type implants. The resulting immobility contributes to the maintenance of a healthy soft tissue seal, and prevents bacterial invasion into the peri-implant tissue. In addition, internal connection-type implants can transform occlusal load into strain that stimulates the surrounding bone through the abutment–implant connection. This strain enhances the amount and quality of peri-implant bone.

Taking these findings into account, clinicians may find it advantageous to select internal conical connection-type implants rather than external connection-type implants, to allow dental implants to function properly as artificial organs with long-term predictability.
